# The Influence of Thermal and Mechanical Aging on the Flexural Properties of Conventional and 3D-Printed Materials Used in Occlusal Splints Manufacturing

**DOI:** 10.3390/ma19020421

**Published:** 2026-01-21

**Authors:** Joanna Smardz, Katarzyna Kresse-Walczak, Heike Meißner, Klaus Böning, Joanna Weżgowiec, Andrzej Małysa, Mieszko Więckiewicz

**Affiliations:** 1 Department of Experimental Dentistry, Wroclaw Medical University, 50-425 Wroclaw, Poland; joanna.smardz@umw.edu.pl (J.S.); joanna.wezgowiec@umw.edu.pl (J.W.); andrzej.malysa@umw.edu.pl (A.M.); 2 Section for Prosthetic Dentistry, Department of Dentistry and Oral Health, Aarhus University, 8000 Aarhus, Denmark; kkresse@dent.au.dk; 3 Department of Prosthodontics, Carl Gustav Carus Faculty of Medicine, Technische Universität Dresden, 01069 Dresden, Germany; heike.meissner@uniklinikum-dresden.de (H.M.); klaus.boening@uniklinikum-dresden.de (K.B.)

**Keywords:** dental materials, dental polymer, intraoral appliance, occlusal splint, PMMA, UDMA, 3D-printed resin, flexural strength, flexural modulus, thermal aging, mechanical aging, three-point bending

## Abstract

**Highlights:**

**What are the main findings?**

- Thermocycling significantly reduced flexural strength in PMMA and SLA-printed resins.
- UDMA-based material stayed unaffected under aging conditions.
- UDMA-based material showed significantly lower values of flexural strength and modulus when compared to PMMA and SLA-printed resins.

**What is the implication of the main findings?**

- Material selection should consider both initial properties and long-term environmental changes.

**Abstract:**

Occlusal splints are a type of intraoral appliance that are widely used for the management of temporomandibular disorders and bruxism, yet limited evidence exists regarding the comparative effects of combined aging on conventional and digitally manufactured materials. This in vitro study evaluated the influence of thermal and mechanical aging on the flexural properties of three materials commonly used for the manufacturing of occlusal devices: self-curing poly(methyl methacrylate) (PMMA, control), light-cured urethane dimethacrylate (UDMA)-based resin, and stereolithography (SLA)-printed photopolymer. Seventy-two standardized specimens (*n* = 24 per material; 64 × 10 × 3.3 mm) were fabricated, then randomly allocated to three groups (*n* = 8): control, thermocycling (10,000 cycles, 5 °C/55 °C), and combined thermocycling with mechanical loading (1000 cycles). Flexural strength and modulus were determined by three-point bending tests and analyzed using a two-way analysis of variance (ANOVA) with Tukey’s post hoc test (α = 0.05). Thermocycling significantly reduced flexural strength in PMMA (65.19 ± 6.68 to 57.94 ± 7.15 MPa) and SLA (67.67 ± 1.54 to 59.37 ± 8.80 MPa) groups (*p* < 0.05), while UDMA group (45.489 ± 3.905 to 43.123 ± 4.367 MPa) demonstrated no significant changes (*p* ≥ 0.05). UDMA exhibited substantially and significantly lower flexural properties compared to PMMA and SLA across all conditions (*p* < 0.0001). Thermal aging slightly compromises the mechanical properties of PMMA and SLA-printed materials, whereas UDMA-based resins exhibit good aging resistance but considerably lower initial values. While UDMA-based resin showed superior aging resistance, its lower baseline mechanical properties may limit its application in high-stress clinical scenarios compared to PMMA and SLA-printed materials. Material selection should consider both initial properties and long-term environmental changes.

## 1. Introduction

Occlusal splints represent an essential element of conservative management for temporomandibular disorders (TMD) and bruxism, conditions that affect a substantial proportion of the adult population worldwide [1,2,3]. Epidemiological studies have consistently reported that TMD affects approximately 5–12% of the general population, with global prevalence estimates reaching 34% when broader diagnostic criteria are applied [2,3]. Similarly, bruxism prevalence ranges from 8% to 31% in adult populations, with significant regional variations [1,4]. Consequently, an estimated 8–12% of adults require some form of occlusal therapy during their lifetime, underscoring the clinical significance of intraoral appliance materials [1,2,3,4,5,6].

The therapeutic efficacy of occlusal splints in managing TMD and bruxism has been well-documented in the literature. Systematic reviews and meta-analyses have demonstrated that rigid stabilization splints produce significant reductions in chronic pain and improvements in mandibular mobility, with high reported improvement rates even when used as monotherapy [7,8,9]. Clinical studies examining long-term outcomes have reported significant symptom remission following splint therapy [10]. Furthermore, protective splints have been shown to potentially influence the risk of restoration failure among patients with bruxism, underscoring the importance of appliance durability for sustained clinical benefit [11].

The mechanical properties of materials used in intraoral appliance fabrication directly influence both therapeutic outcomes and clinical longevity [12,13]. Flexural strength and flexural modulus represent critical parameters that determine an appliance’s ability to withstand masticatory forces, resist deformation, and maintain dimensional stability throughout the treatment period [14,15]. The complex and dynamic oral environment subjects these materials to various degradation mechanisms, including thermal, mechanical, chemical and biological degradation [16,17,18].

Poly(methyl methacrylate) (PMMA) has long been the gold standard for denture base and splint fabrication, owing to its favorable processing characteristics, acceptable mechanical properties, and established clinical track record [19,20,21,22]. The emergence of urethane dimethacrylate (UDMA)-based resins has provided clinicians with alternative materials characterized by distinct polymerization chemistry and cross-linking characteristics [23,24]. UDMA monomers form highly cross-linked networks upon photopolymerization, potentially conferring enhanced resistance to hydrolytic degradation [25]. The flexible urethane linkages within the polymer backbone may provide improved toughness characteristics, although this structural feature can also result in reduced elastic modulus compared to PMMA-based materials [26]. Recent advances in digital dentistry have introduced additive manufacturing technologies, particularly stereolithography (SLA), as viable alternatives for occlusal devices fabrication [27,28]. Some of SLA-printed photopolymers are specifically formulated for long-term intraoral applications and have received regulatory clearance for occlusal splint production. Comparative studies have demonstrated that contemporary 3D-printed materials can be able to achieve mechanical properties comparable to or exceeding those of conventional autopolymerizing resins, although significant variability exists among different printing systems and materials [29,30,31]. Several studies demonstrate that additive manufacturing parameters, such as printing orientation and layer thickness, significantly influence the mechanical behavior and aging response of 3D-printed materials. Farkas et al. observed that thinner layers generally yielded higher tensile values [32]. In a recent in vitro investigation, Mudhaffer et al. reported that print orientation modulates flexural strength and modulus of resin materials indicated for definitive and interim dental restorations, with specimens printed at 90° exhibiting superior mechanical properties across multiple artificial aging time points (water storage at 37 °C), suggesting that orientation influences susceptibility to degradative processes [33]. Broader evidence from systematic analyses confirms that orientation affects various physical properties of 3D-printed polymeric dental restorations, including mechanical parameters that are known to deteriorate under artificial aging (e.g., water absorption, color stability, surface morphology), further supporting the need to consider these manufacturing variables in studies of long-term performance [34].

The influence of artificial aging on dental polymer properties has been extensively investigated using thermocycling protocols [35,36]. The standard thermocycling regimen (5 °C/55 °C) induces repeated thermal expansion and contraction cycles that can promote water absorption, polymer chain relaxation, and microcrack formation [35,36,37]. Studies examining PMMA-based materials have consistently reported significant reductions in flexural strength following thermocycling [38,39].

Mechanical fatigue is an additional degradation mechanism that may compromise material integrity, either independently or synergistically with thermal aging [40,41]. Cyclic loading may simulate the repetitive masticatory forces transmitted through occlusal splints during normal function and parafunctional activities [42]. It has been reported that cumulative mechanical damage can significantly reduce material longevity [43,44,45].

Despite extensive research on individual aging mechanisms, limited evidence exists regarding the combined effects of thermal and mechanical aging on materials explicitly used for the fabrication of occlusal devices. Furthermore, comparative data examining the aging behavior of conventional PMMA, UDMA-based resins, and 3D-printed photopolymers under standardized conditions remain scarce. Such information is essential for evidence-based material selection and for establishing realistic expectations regarding appliance longevity in clinical practice.

Therefore, the aim of this in vitro study was to evaluate the influence of thermal aging alone and combined thermal-mechanical aging on the flexural strength and flexural modulus of three materials commonly used for occlusal splints fabrication. The null hypotheses tested were: (1) artificial aging does not significantly affect the mechanical properties of the materials investigated, and (2) there are no significant differences among materials in their response to aging protocols.

## 2. Materials and Methods

### 2.1. Materials

The in vitro study presented evaluated three materials used to fabricate occlusal devices. These materials were selected based on their different chemical composition and manufacturing methodologies, as well as broad clinical application as reported in contemporary dental literature [46]:

- Classic self-curing poly(methyl methacrylate) (PMMA) resin (PMMA group, Estetic Ort; Wiedent, Łódź, Poland) representing the conventional approach to splints manufacturing This material consists of a powder and liquid component.
- Light-cured UDMA-based resin (UDMA group, Durasplint LC; Scheu Dental, Iserlohn, Germany). This material is supplied as pre-formed bars and consists primarily of UDMA matrix with minor additions of acrylic resin, photoinitiators, and crosslinkers.
- Photopolymer designed explicitly for 3D printing using SLA technology (SLA group, Dental LT Clear V1; Vertex Dental, Soesterberg, The Netherlands). This material is classified as a Class IIa biocompatible resin primarily composed of methacrylate oligomers, methacrylate monomers, and photoinitiators.

### 2.2. Preparation of Specimens

A total of 72 standardized specimens of rectangular shape were fabricated for this study (*n* = 24 from each material). The specimen dimensions (64 mm × 10.0 (±0.2) mm × 3.3 (±0.2) mm) were selected following the recommendations documented in similar investigations and PN-EN ISO 20795-1:2013 guidelines [46,47].

The PMMA specimens were prepared using a standardized compression molding technique with standardized metal molds to ensure dimensional accuracy [30,48,49]. The material was mixed according to the instructions of the manufacturer following powder-to-liquid ratio standardized at 3:1 by weight, packed into the molds at the dough-like stage, compressed between glass plates under 2.0 bar pressure, and allowed to polymerize for 20 min at room temperature (23 ± 1 °C), followed by 30 min in a pressure pot (Polyclav; Dentaurum, Ispringen, Germany) at 55 °C and pressure of 2.5 bar [49].

The UDMA-based specimens were prepared by compression molding using calibrated silicone molds to ensure dimensional accuracy. The specimens were then polymerized in a laboratory light-curing unit (LC-6 Light Oven; Scheu Dental, Iserlohn, Germany) for 2 × 10 min, with specimens inverted halfway through the process to ensure complete polymerization of both surfaces [46].

The SLA-printed specimens were designed using Meshmixer version 3.5.474 (Autodesk, San Francisco, CA, USA). Adding supports and setting printing parameters was made using PreForm software ver. 3.28.1 (Formlabs Inc., Somerville, MA, USA). A Form 2 printer (Formlabs) was used to print specimens at a resolution of 100 μm, and 90° printing orientation. When printed, the specimens were washed 2 × 10 min in 99% isopropanol (PPH STANLAB, Lublin, Poland), air-dried at room temperature for 30 min and postcured in a Form Cure (Formlabs) at 80 °C for 20 min. Finally, the supports were removed by cutting and grinding [46].

Following fabrication, all specimens were carefully inspected for defects such as porosities, inclusions, or dimensional irregularities to exclude and replace specimens exhibiting any visible defects. The specimen edges were polished with 1000-grit silicon carbide paper (P.S. Trading, Oltarzew, Poland) to remove processing artifacts. The water sandpaper (grit P500, P1000, P1200, P.S. Trading, Oltarzew, Poland) and 0.6 mm pumice stone powder (Everall7, Warsaw, Poland) were used to finish the specimens polishing paste for resin and metals (Everall7, Warsaw, Poland) was used to polish the upper side of each specimen using POLIRET Mini (REITEL Feinwerktechnik GmbH, Bad Essen, Germany) for 1 min. Finally, each specimen was rinsed under water [46] and underwent ultrasonic cleaning in distilled water with a mild detergent (Clean and clever, IGEFA Handelsgesellschaft mbH & Co. KG, Ahrensfelde, Germany) at 30 °C for 10 min (Elmasonic S30H; Elma Schmidbauer GmbH, Singen, Germany).

After that, the specimens of each material underwent a random division into three equal groups (*n* = 8 per group) according to the subsequent exposure: (1) no exposure (control group); (2) thermocycling of 10,000 cycles; (3) thermocycling of 10,000 cycles and cyclic loading of 1000 cycles.

### 2.3. Thermal Aging

A thermocycler (THE-1200; SD Mechatronik GmbH, Feldkirchen-Westerham, Germany) was used to simulate thermal aging with cyclical exposure to temperature-controlled distilled water (5 °C/55 °C). Each cycle lasted 77 s, with 27 s of immersion time in each water container and transfer time of 9 s. A total of 10,000 cycles were performed to simulate 12 months of use [48].

### 2.4. Mechanical Aging

The mechanical aging was conducted through sinusoidal alternating load (Universal Testing Machine Inspekt-Micro LC 100 N; Hegewald & Peschke, Meß- und Prüftechnik GmbH, Nossen, Germany) and resembled a cyclic three-point bending test in temperature-controlled artificial saliva at 37 °C (UKD saliva solution; University Pharmacy, Dresden, Germany). A point load (stainless steel ball Ø 10 mm) was applied to the specimen centrally. A frequency of 1 Hz, with a displacement of 2 mm, vertical deflection of the specimen under the load of 1 mm, a loading speed of 1 mm/s, and a preload of 1 N were used to perform bending cycles. A total of 1000 cycles were performed, which roughly correspond to 1 year of wear [48].

### 2.5. Flexural Properties Evaluation

Before testing, the specimens were conditioned in distilled water at 37 °C for 50 h. A Magnusson digital caliper (150 mm) (Limit, Alingsås, Sweden) was used to measure height and width of each specimen at 5 points. Immediately before testing, the mean cross-sectional area was calculated. The three-point bending test was performed using the Universal Testing Machine (Z10-X700; AML Instruments Ltd., Lincoln, UK) at a constant displacement rate of 5 mm/min and a span length of 50 mm between the supports [46,47]. The tests were conducted by a single investigator in accordance with the PN-EN ISO 20795-1:2013 guidelines [47].

Flexural strength (σ [MPa]) was calculated using the following formula (Equation (1)):

σ = 3Fl/2bh^2^ (1)

where F—maximum load [N]; l—distance between the supports [mm] (±0.01 mm); b—width of the specimen [mm]; and h—height of the specimen [mm].

Flexural modulus (E [MPa]) was determined using the following formula (Equation (2)):

E = (F/d)/(l^3^/[4bh^3^]) (2)

where load (F) divided by displacement (d) is the slope in the linear elastic region of the load/displacement curve; l, b and h are as defined above [46].

### 2.6. Statistical Analysis

Statistical analysis was performed using GraphPad Prism version 9.0 (GraphPad Software Inc., San Diego, CA, USA). All data were expressed as mean ± standard deviation, and as box plots presenting the five-number summary of a dataset (minimum, first quartile, median, third quartile, and maximum). The normality of data distribution was verified using the Shapiro–Wilk test. All the data were normally distributed. A two-way analysis of variance (ANOVA) was employed to assess the effects of material type and aging method, as well as their interaction, on flexural strength and flexural modulus. When significant effects were detected, Tukey’s honestly significant difference post hoc test was applied for multiple pairwise comparisons. The level of statistical significance was set at α = 0.05 for all analyses.

## 3. Results

**Figure 1:**
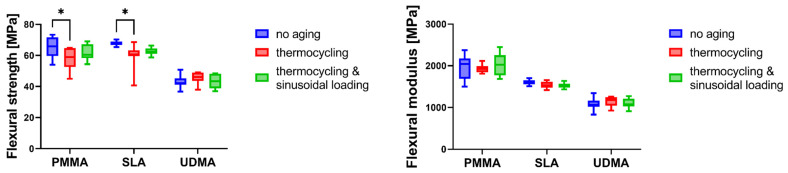
Comparison of flexural strength and flexural modulus between three materials subjected to various methods of artificial aging; asterisks indicate significant differences between methods of aging within each material type (two-way ANOVA with post hoc Tukey’s multiple comparisons test, * *p* < 0.05).

The flexural strength and flexural modulus values for all materials and aging conditions are presented in Table 1 and illustrated in Figure 1.

### 3.1. Effect of Aging on Flexural Strength

Within-group comparisons revealed a distinctive pattern in the response to aging protocols. Thermocycling alone significantly reduced flexural strength in both PMMA and SLA groups compared to baseline values (*p* < 0.05 for both comparisons). The PMMA group demonstrated a decrease from 65.19 ± 6.68 MPa to 57.94 ± 7.15 MPa. Similarly, the SLA group exhibited a reduction from 67.67 ± 1.54 MPa to 59.37 ± 8.80 MPa. Notably, the combined thermocycling and mechanical loading protocol yielded intermediate values that did not differ significantly from baseline in either PMMA (61.53 ± 5.11 MPa, *p* ≥ 0.05) or SLA (62.55 ± 2.42 MPa, *p* ≥ 0.05) groups. In contrast, the UDMA group demonstrated stability across all aging conditions (*p* ≥ 0.05 for all comparisons) (Table 1, Figure 1).

### 3.2. Effect of Aging on Flexural Modulus

The flexural modulus data revealed no significant within-group differences for any material following aging protocols (*p* ≥ 0.05 for all comparisons) (Table 1, Figure 1).

### 3.3. Inter-Material Comparisons

Between-group comparisons revealed highly consistent patterns across all aging conditions (Table 2 and Table 3).

For flexural strength, PMMA and SLA groups showed no statistical differences under all conditions (*p* > 0.05). UDMA demonstrated significantly lower flexural strength compared to both PMMA and SLA groups across all aging groups (*p* < 0.0001 in all comparisons). The magnitude of these differences was substantial: Flexural strength of PMMA exceeded UDMA by 12.45 to 22.06 MPa, while SLA exceeded UDMA by 13.88 to 24.54 MPa, depending on aging condition (Table 2).

For flexural modulus, all inter-material comparisons achieved statistical significance (*p* < 0.0001). A consistent hierarchy was observed: PMMA > SLA > UDMA across all conditions. The mean difference of flexural modulus between PMMA and UDMA ranged from 790.1 to 937.5 MPa, while the difference between PMMA and SLA ranged from 362.5 to 515.4 MPa, and between SLA and UDMA from 399.0 to 531.9 MPa (Table 3).

## 4. Discussion

The present study investigated the effects of thermal and mechanical aging on the flexural properties of three materials used for occlusal splints fabrication. The results demonstrated material-specific responses to aging protocols, with thermocycling alone significantly reducing flexural strength in PMMA and SLA groups, while UDMA group exhibited stability under all tested conditions. These findings lead to partial rejection of the first null hypothesis, as aging significantly affected the mechanical properties of specific materials under specific conditions. The second null hypothesis was rejected, as significant differences among materials were observed both in baseline properties and in their response to aging protocols. A consistent hierarchy was observed: PMMA > SLA > UDMA for flexural modulus; PMMA and SLA > UDMA for flexural strength across all conditions.

The significant reduction in flexural strength observed in PMMA following thermocycling is consistent with previous investigations examining the effects of thermal aging on acrylic resins [38,39]. This degradation can be attributed to several interconnected mechanisms operating at the molecular level. Water absorption during thermal cycling causes plasticization of the polymer matrix, with water molecules diffusing between polymer chains and disrupting intermolecular hydrogen bonding [50,51,52]. The repeated thermal expansion and contraction cycles induce internal stresses that may exceed the material’s yield strength locally, promoting microcrack initiation and propagation [53]. The SLA-printed resin demonstrated a similar pattern of degradation following aging, which aligns with recent literature on 3D-printed dental materials [54,55]. The layer-by-layer manufacturing process inherent to SLA technology creates interfaces between successive layers that may act as preferential pathways for water penetration and stress concentration [56,57]. Despite post-curing protocols designed to maximize degree of conversion, residual unreacted monomers may leach from the polymer network when exposed to aqueous environments, creating voids that compromise mechanical integrity [58]. The stability of UDMA-based material under thermal and mechanical aging conditions represents a noteworthy finding with important clinical implications. UDMA forms highly cross-linked networks through photopolymerization, with the urethane linkages providing both flexibility and hydrolytic resistance [25,59]. Previous investigations have reported that UDMA-based dental materials achieve degrees of conversion higher than those observed in other systems [24,60]. This enhanced conversion results in a more completely polymerized network with fewer unreacted methacrylate groups susceptible to hydrolysis. Additionally, the cross-linked architecture restricts polymer chain mobility and limits water diffusion into the bulk material [61].

Thermocycling exposes polymer-based dental materials to repeated temperature fluctuations in an aqueous environment, which can accelerate water uptake and thermo-mechanical stress development. For PMMA-based materials, absorbed water is widely described as a plasticizer that increases free volume and chain mobility, promotes polymer chain relaxation, and can reduce resistance to crack initiation/propagation under bending. This mechanism is consistent with our results, where PMMA showed a measurable decrease in flexural strength after thermocycling (≈−11%), while the flexural modulus changed only marginally (≈−2%). Similar findings have been reported for denture base and resin-based polymers subjected to thermocycling, where flexural properties decrease as a consequence of water–polymer interactions and thermal fatigue [62,63].

In contrast, the comparatively stable performance of the UDMA-based material in our study (for flexural strength ≈+6% after thermocycling and ≈+0.4% after combined aging) can be explained by the characteristics of UDMA networks. UDMA-containing materials form cross-linked polymer structures that restrict long-range segmental motion, thereby limiting water-induced softening and delaying relaxation-driven loss of strength. Structural property analyses of dental UDMA-based materials emphasize the key role of conversion/crosslink density and intermolecular interactions in controlling mechanical stability and water sorption–related effects. Moreover, experimental aging studies on UDMA networks show that water uptake may follow diffusion-governed behavior and that hydrolytic aging is strongly governed by polymer chemistry and network architecture [24,64,65].

Finally, the SLA 3D-printed resin also demonstrated a reduction in flexural strength after thermocycling (≈−12%), which aligns with the broader additive-manufacturing literature showing that such materials can present higher water sorption/solubility and measurable property shifts after thermal cycling. This may be related to factors such as residual monomer, degree of conversion, and microstructural heterogeneity (including interlayer interfaces) that can facilitate water diffusion and aging-related weakening [66,67].

The finding that combined thermal and mechanical aging did not differ significantly from baseline values, while thermocycling alone produced a significant change initially appears counterintuitive, as cumulative damage from sequential stress exposures would be expected. However, several plausible mechanisms may explain this phenomenon. The first hypothesis involves strain-induced structural reorganization. Cyclic mechanical loading may induce microstructural changes that partially compensate for thermal degradation, a phenomenon recognized in polymer science as strain hardening or stress-induced chain alignment. Repetitive bending cycles could promote reorganization of polymer chains into more mechanically favorable orientations, effectively counteracting the disruptive effects of thermal cycling on the polymer network [40,41,68,69]. However, the strain hardening hypothesis is extrapolated from general polymer physics literature and has not been directly validated for occlusal splint materials under the specific loading conditions employed in this study. Future research should directly investigate polymer chain reorganization in dental resin materials subjected to combined thermal-mechanical aging protocols as well as to mechanical aging alone, for comparative purposes. Alternatively, the water absorption occurring during thermocycling may have induced plasticization effects that allowed the polymer matrix to accommodate subsequent mechanical stresses more readily, reducing stress concentration at defect sites [25,63,70]. On the other hand, taking into consideration the explorative character of our work, the results obtained could be the effect of small sample size that could influence not detecting the difference between baseline and aging conditions.

When comparing the present results to requirements for denture base polymers, important distinctions emerge among materials. The standard minimum flexural strength of 65 MPa and flexural modulus of 2000 MPa for Type 1 (heat-polymerized) and Type 4 (light-activated) materials, with slightly relaxed requirements for Type 2 (autopolymerized) materials (60 MPa and 1500 MPa, respectively) were defined [47]. In the control (non-aged) group, both PMMA (65.19 ± 6.68 MPa) and SLA (67.67 ± 1.54 MPa) met or exceeded flexural strength requirement for the Type 1 and 4 materials, while UDMA (43.12 ± 4.37 MPa) fell substantially below this threshold. Following thermocycling, neither PMMA (57.94 ± 7.15 MPa) nor SLA (59.37 ± 8.80 MPa) maintained compliance with Type 1 requirements, although both remained close to the Type 2 threshold. However, it should be noted that PMMA values of flexural strength in control group obtained in this study were lower than those reported in the literature [71]. Regarding flexural modulus, only PMMA specimens achieved values exceeding 2000 MPa, and this occurred only in the combined aging group (2036.83 ± 263.60 MPa). SLA and UDMA materials demonstrated consistently lower modulus values across all conditions, ranging from 1077.41 to 1609.32 MPa. While lower elastic modulus may provide some advantages in terms of stress distribution and patient comfort, excessively flexible appliances may fail to provide adequate occlusal stabilization or protection against parafunctional forces, as well as they will be less resistant to deformation [10,14,15].

The flexural strength observed between PMMA and SLA materials across all conditions did not differ significantly. This supports the clinical viability of 3D-printed intraoral appliances. Recent systematic reviews suggest that contemporary additive manufacturing technologies can produce dental devices with mechanical properties equivalent to or exceeding those of conventional fabrication methods [29,30,72]. In contrast, Benli et al. reported that 3D-printed splint materials should not be considered as the primary choice for long-term treatments due to their low mechanical and chemical properties [73]. The digital workflow associated with SLA printing offers additional advantages, including improved dimensional accuracy, reduced laboratory time, and enhanced reproducibility compared to manual fabrication techniques [74]. Summarizing, the statistical similarity between PMMA and SLA flexural strength across all aging conditions represents an important finding for clinical practice. The choice between conventional PMMA and SLA-printed resin should therefore be guided by other factors, including fabrication workflow, dimensional accuracy, cost-effectiveness, and aesthetic requirements, rather than flexural strength considerations.

The significantly lower flexural strength of UDMA was consistent across all conditions. It presents both advantages and limitations for clinical application. The urethane linkages provide both flexibility and enhanced hydrolytic resistance compared to the ester linkages predominant in PMMA, while the high degree of conversion results in fewer unreacted functional groups susceptible to degradation [75]. However, the stability of UDMA comes at the cost of substantially lower absolute mechanical properties. The clinical decision therefore involves a trade-off: PMMA and SLA offer higher initial strength but with potential for some degradation over time (and in the case of SLA, with greater unpredictability), while UDMA offers substantially lower but consistent properties throughout the service life. To overcome this disadvantage, another option which could be applied includes the combination of the UDMA-based material, which is considered as an adjustment, with the base fabricated from thermoformed material, e.g., polyethylene terephthalate glycol (PET-G). This could potentially improve the low mechanical properties of UDMA. Our previous research provided evidence regarding superior chemical stability of such combination over conventional heat-activated PMMA and 3D-printable Dental LT clear resin [75]. As biological risks associated with the leakage of compounds upon long-term interaction of dental materials with the complex intraoral environment must be minimized, such observations should also be considered in the process of material selection.

Several limitations of the present study should be acknowledged. The simplified rectangular specimen geometry does not replicate the complex three-dimensional architecture of clinical intraoral appliances [76]. The aging protocols did not incorporate salivary enzymes, pH variations, or biofilm formation [17,77]. The mechanical loading protocol (1000 cycles) may not adequately simulate cumulative fatigue in patients with severe parafunctional habits [78]. Moreover, cyclic loading tests were reported to require more standardized guidelines for testing and reporting; thus, it should be clarified that current standards and the available literature do not define a universally accepted number of cycles for fatigue-based artificial aging of dental polymer materials [79]. The lack of complementary microstructural and chemical characterization before and after aging could also be considered as limitation of presented study. The scanning electron microscopy and Fourier transform infrared spectroscopy could have provided direct evidence of aging-related morphological changes (e.g., microcracks/porosity) and polymer network alterations, enabling a more mechanistic explanation of the observed mechanical property changes. Finally, only single representatives from each material category were evaluated.

## 5. Conclusions

Thermocycling alone significantly reduced flexural strength in both PMMA and SLA-printed materials. UDMA-based resin exhibited stability under all aging protocols but with substantially lower absolute flexural strength. Only PMMA specimens approached Type 1 requirements for flexural modulus (≥2000 MPa). While UDMA-based resin showed superior aging stability, its lower baseline mechanical properties may limit its application in high-stress clinical scenarios compared to PMMA and SLA-printed materials. Results obtained in this study suggest that material selection for occlusal splints should consider both the specific mechanical demands of the clinical application and the expected duration of service. Considering flexural strength and modulus, PMMA still seems to be material of choice for occlusal splints fabrication.

## Figures and Tables

**Table 1:** Flexural strength and flexural modulus of three materials subjected to various methods of artificial aging (mean ± SD).

Materials	Flexural Strength [MPa]					Flexural Modulus [MPa]				
	No Aging (Mean ± SD)	Thermocycling (Mean ± SD)	Δ% vs. No Aging	Thermocycling & Sinusoidal Loading (Mean ± SD)	Δ% vs. No Aging	No Aging (Mean ± SD)	Thermocycling (Mean ± SD)	Δ% vs. No Aging	Thermocycling & Sinusoidal Loading (Mean ± SD)	Δ% vs. No Aging
PMMA	65.19 ± 6.68	57.94 ± 7.15	−11.11%	61.53 ± 5.11	−5.61%	1971.85 ± 292.35	1930.66 ± 101.29	−2.09%	2036.83 ± 263.60	+3.30%
SLA	67.67 ± 1.54	59.37 ± 8.80	−12.25%	62.55 ± 2.42	−7.56%	1609.32 ± 61.95	1539.58 ± 81.46	−4.33%	1521.45 ± 63.95	−5.46%
UDMA	43.12 ± 4.37	45.49 ± 3.90	+5.49%	43.31 ± 4.59	+0.44%	1077.40 ± 155.47	1140.58 ± 120.57	+5.86%	1099.36 ± 116.50	+2.04%

**Table 2:** Differences in flexural strength [MPa] between materials grouped by the method of aging applied—results of two-way ANOVA with post hoc Tukey’s multiple comparisons test; SE—standard error, CI—confidence interval, q—the test statistic, df—the number of degrees of freedom, ns—not significant, ****—*p* < 0.0001.

Aging	Materials	Mean Difference	SE	95.00% CI	q	df	Summary	*p*-Value
no aging	PMMA vs. SLA	−2.480	2.673	−8.900 to 3.940	1.312	61.00	ns	0.6249
	PMMA vs. UDMA	22.06	2.766	15.42 to 28.71	11.28	61.00	****	<0.0001
	SLA vs. UDMA	24.54	2.766	17.90 to 31.19	12.55	61.00	****	<0.0001
thermocycling	PMMA vs. SLA	−1.429	2.766	−8.075 to 5.217	0.7306	61.00	ns	0.8636
	PMMA vs. UDMA	12.45	2.673	6.034 to 18.88	6.590	61.00	****	<0.0001
	SLA vs. UDMA	13.88	2.766	7.238 to 20.53	7.097	61.00	****	<0.0001
thermocycling & sinusoidal loading	PMMA vs. SLA	−1.017	2.673	−7.438 to 5.403	0.5383	61.00	ns	0.9233
	PMMA vs. UDMA	18.22	2.673	11.80 to 24.64	9.640	61.00	****	<0.0001
	SLA vs. UDMA	19.24	2.673	12.81 to 25.66	10.18	61.00	****	<0.0001

**Table 3:** Differences in flexural modulus [MPa] between materials grouped by the method of aging applied—results of two-way ANOVA with post hoc Tukey’s multiple comparisons test; SE—standard error, CI—confidence interval, q—the test statistic, df—the number of degrees of freedom, ****—*p* < 0.0001.

Aging	Materials	Mean Difference	SE	95.00% CI	q	df	Summary	*p*-Value
no aging	PMMA vs. SLA	362.5	80.72	168.6 to 556.4	6.351	61.00	****	<0.0001
	PMMA vs. UDMA	894.4	83.56	693.7 to 1095	15.14	61.00	****	<0.0001
	SLA vs. UDMA	531.9	83.56	331.2 to 732.6	9.003	61.00	****	<0.0001
thermocycling	PMMA vs. SLA	391.1	83.56	190.4 to 591.8	6.619	61.00	****	<0.0001
	PMMA vs. UDMA	790.1	83.56	589.4 to 990.8	13.37	61.00	****	<0.0001
	SLA vs. UDMA	399.0	80.72	205.1 to 592.9	6.990	61.00	****	<0.0001
thermocycling & sinusoidal loading	PMMA vs. SLA	515.4	80.72	321.5 to 709.3	9.029	61.00	****	<0.0001
	PMMA vs. UDMA	937.5	80.72	743.6 to 1131	16.42	61.00	****	<0.0001
	SLA vs. UDMA	422.1	80.72	228.2 to 616.0	7.395	61.00	****	<0.0001

## Data Availability

The original contributions presented in this study are included in the article. Further inquiries can be directed to the corresponding author.

## Abbreviations

The following abbreviations are used in this manuscript:

PMMA	poly(methyl methacrylate)
PET-G	polyethylene terephthalate glycol
SLA	stereolithography
TMD	temporomandibular disorders
UDMA	urethane dimethacrylate

## References

[1] Zieliński G., Pająk A., Wójcicki M. (2024). Global Prevalence of Sleep Bruxism and Awake Bruxism in Pediatric and Adult Populations: A Systematic Review and Meta-Analysis. J. Clin. Med..

[2] Zieliński G., Pająk-Zielińska B., Ginszt M. (2024). A Meta-Analysis of the Global Prevalence of Temporomandibular Disorders. J. Clin. Med..

[3] Ohrbach R., Dworkin S.F. (2016). The Evolution of TMD Diagnosis: Past, Present, Future. J. Dent. Res..

[4] Lobbezoo F., Ahlberg J., Raphael K.G., Wetselaar P., Glaros A.G., Kato T., Santiago V., Winocur E., De Laat A., De Leeuw R. (2018). International Consensus on the Assessment of Bruxism: Report of a Work in Progress. J. Oral Rehabil..

[5] Zhang S.H., He K.X., Lin C.J., Liu X.D., Wu L., Chen J., Rausch-Fan X. (2020). Efficacy of occlusal splints in the treatment of temporomandibular disorders: A systematic review of randomized controlled trials. Acta Odontol. Scand..

[6] Al-Moraissi E.A., Farea R., Qasem K.A., Al-Wadeai M.S., Al-Sabahi M.E., Al-Iryani G.M. (2020). Effectiveness of occlusal splint therapy in the management of temporomandibular disorders: Network meta-analysis of randomized controlled trials. Int. J. Oral Maxillofac. Surg..

[7] Fricton J., Look J.O., Wright E., Alencar F.G.P., Chen H., Lang M., Ouyang W., Velly A.M. (2010). Systematic Review and Meta-Analysis of Randomized Controlled Trials Evaluating Intraoral Orthopedic Appliances for Temporomandibular Disorders. J. Orofac. Pain.

[8] Kuzmanovic Pficer J., Dodic S., Lazic V., Trajkovic G., Milic N., Milicic B. (2017). Occlusal Stabilization Splint for Patients with Temporomandibular Disorders: Meta-Analysis of Short and Long Term Effects. PLoS ONE.

[9] Orzeszek S., Waliszewska-Prosol M., Ettlin D., Seweryn P., Straburzynski M., Martelletti P., Jenca A., Wieckiewicz M. (2023). Efficiency of Occlusal Splint Therapy on Orofacial Muscle Pain Reduction: A Systematic Review. BMC Oral Health.

[10] Seifeldin S.A., Elhayes K.A. (2015). Soft versus Hard Occlusal Splint Therapy in the Management of Temporomandibular Disorders (TMDs). Saudi Dent. J..

[11] Granell-Ruiz M., Fons-Font A., Labaig-Rueda C., Martínez-González A., Román-Rodríguez J.L., Solá-Ruiz M.F. (2010). A Clinical Longitudinal Study 323 Porcelain Laminate Veneers. Period of Study from 3 to 11 Years. Med. Oral Patol. Oral Cir. Bucal.

[12] Nassif M., Haddad C., Habli L., Zoghby A. (2023). Materials and manufacturing techniques for occlusal splints: A literature review. J. Oral Rehabil..

[13] Soliman T.A., Raffat E.M., Farahat D.S. (2023). Evaluation of Mechanical Behavior of CAD/CAM Polymers for Long-term Interim Restoration Following Artificial Aging. Eur. J. Prosthodont. Restor. Dent..

[14] Cui X., Li F., Jiang J. (2025). Effects of simulated intraoral temperatures and wet environments on the stress relaxation properties of thermoplastic aligner materials. Head Face Med..

[15] Prpic V., Spehar F., Stajdohar D., Bjelica R., Cimic S., Par M. (2023). Mechanical Properties of 3D-Printed Occlusal Splint Materials. Dent. J..

[16] Ferracane J.L. (2006). Hygroscopic and Hydrolytic Effects in Dental Polymer Networks. Dent. Mater..

[17] Finer Y., Santerre J.P. (2004). Salivary Esterase Activity and Its Association with the Biodegradation of Dental Composites. J. Dent. Res..

[18] Asmussen E., Peutzfeldt A. (2001). Influence of Selected Components on Crosslink Density in Polymer Structures. Eur. J. Oral Sci..

[19] El-Din M.S., Ellakany P., Fouda S.M., Gad M.M. (2025). Effect of various beverages on the surface roughness and color stability of different denture base resins: An in vitro study. Dent. Med. Probl..

[20] Fortes C.V., Ribeiro A.B., De Wever B., Sakly A., Oliveira V.C., Clemente L.M., Watanabe E., Silva-Lovato C.H. (2025). Evaluation of biofilm formation, adhesive strength and effectiveness of cleaning protocols on adhesive-containing acrylic resin specimens: An in vitro study. Dent. Med. Probl..

[21] Zafar M.S. (2020). Prosthodontic Applications of Polymethyl Methacrylate (PMMA): An Update. Polymers.

[22] Alla R.K., Swamy R.K.N., Vyas R., Konakarchi A. (2015). Conventional and Contemporary Polymers for the Fabrication of Denture Prosthesis: Part I—Overview, Composition and Properties. Int. J. Appl. Dent. Sci..

[23] Kanie T., Kadokawa A., Arikawa H., Fujii K., Ban S. (2010). Flexural properties of ethyl or methyl methacrylate-UDMA blend polymers. Dent. Mater. J..

[24] Szczesio-Wlodarczyk A., Domarecka M., Kopacz K., Sokolowski J., Bociong K. (2021). An Evaluation of the Properties of Urethane Dimethacrylate-Based Dental Resins. Materials.

[25] Sideridou I.D., Tserki V., Papanastasiou G. (2003). Study of Water Sorption, Solubility and Modulus of Elasticity of Light-Cured Dimethacrylate-Based Dental Resins. Biomaterials.

[26] Lin C.H., Lin Y.M., Lai Y.L., Lee S.Y. (2020). Mechanical Properties, Accuracy, and Cytotoxicity of UV-Polymerized 3D Printing Resins Composed of Bis-EMA, UDMA, and TEGDMA. J. Prosthet. Dent..

[27] Tian Y., Chen C., Xu X., Wang J., Hou X., Li K., Lu X., Shi H., Lee E.S., Jiang H.B. (2021). A Review of 3D Printing in Dentistry: Technologies, Affecting Factors, and Applications. Scanning.

[28] Rezaie F., Farshbaf M., Dahri M., Masjedi M., Maleki R., Amini F., Wirth J., Moharamzadeh K., Weber F.E., Tayebi L. (2023). 3D Printing of Dental Prostheses: Current and Emerging Applications. J. Compos. Sci..

[29] Maleki T., Meinen J., Coldea A., Reymus M., Edelhoff D., Stawarczyk B. (2024). Mechanical and Physical Properties of Splint Materials for Oral Appliances Produced by Additive, Subtractive and Conventional Manufacturing. Dent. Mater..

[30] Prpić V., Schauperl Z., Ćatić A., Dulčić N., Čimić S. (2020). Comparison of Mechanical Properties of 3D-Printed, CAD/CAM, and Conventional Denture Base Materials. J. Prosthodont..

[31] van Noort R. (2012). The future of dental devices is digital. Dent. Mater..

[32] Farkas A.Z., Galatanu S.V., Nagib R. (2023). The Influence of Printing Layer Thickness and Orientation on the Mechanical Properties of DLP 3D-Printed Dental Resin. Polymers.

[33] Mudhaffer S., Haider J., Satterthwaite J., Silikas N. (2025). Effects of print orientation and artificial aging on the flexural strength and flexural modulus of 3D printed restorative resin materials. J. Prosthet. Dent..

[34] Alqarawi F.K. (2025). The Influence of Printing Orientation on the Properties of 3D-Printed Polymeric Provisional Dental Restorations: A Systematic Review and Meta-Analysis. J. Funct. Biomater..

[35] Szczesio-Wlodarczyk A., Fronczek M., Ranoszek-Soliwoda K., Grobelny J., Sokolowski J., Bociong K. (2022). The First Step in Standardizing an Artificial Aging Protocol for Dental Composites—Evaluation of Basic Protocols. Molecules.

[36] Gale M.S., Darvell B.W. (1999). Thermal Cycling Procedures for Laboratory Testing of Dental Restorations. J. Dent..

[37] Ghavami-Lahiji M., Firouzmanesh M., Bagheri H., Jafarzadeh Kashi T.S., Razazpour F., Behroozibakhsh M. (2018). The effect of thermocycling on the degree of conversion and mechanical properties of a microhybrid dental resin composite. Restor. Dent. Endod..

[38] Polychronakis N., Sarafianou A., Zissis A., Papadopoulos T. (2017). The Influence of Thermocycling on the Flexural Strength of a Polyamide Denture Base Material. Acta. Stomatol. Croat..

[39] Temizci T., Bozoğulları H.N. (2024). Effect of Thermal Cycling on the Flexural Strength of 3-D Printed, CAD/CAM Milled and Heat-Polymerized Denture Base Materials. BMC Oral Health.

[40] Drummond J.L. (2008). Degradation, Fatigue, and Failure of Resin Dental Composite Materials. J. Dent. Res..

[41] Arola D. (2017). Fatigue Testing of Biomaterials and Their Interfaces. Dent. Mater..

[42] Lin J., Sun M., Zheng Z., Shinya A., Han J., Lin H., Zheng G., Shinya A. (2013). Effects of rotating fatigue on the mechanical properties of microhybrid and nanofiller-containing composites. Dent. Mater. J..

[43] Lohbauer U., Petschelt A., Greil P. (2002). Lifetime Prediction of CAD/CAM Dental Ceramics. J. Biomed. Mater. Res..

[44] Ausiello P., Ciaramella S., Fabianelli A., Gloria A., Martorelli M., Lanzotti A., Watts D.C. (2017). Mechanical Behavior of Bulk Direct Composite versus Block Composite and Lithium Disilicate Indirect Class II Restorations by CAD-FEM Modeling. Dent. Mater..

[45] Rayyan M.M., Aboushelib M., Sayed N.M., Ibrahim A., Jimbo R. (2015). Comparison of Interim Restorations Fabricated by CAD/CAM with Those Fabricated Manually. J. Prosthet. Dent..

[46] Weżgowiec J., Małysa A., Więckiewicz M. (2025). How Does Artificial Aging Affect the Mechanical Properties of Occlusal Splint Materials Processed via Various Technologies?. Dent. Med. Probl..

[47] (2013). Dentistry—Base Polymers—Part 1: Denture Base Polymers.

[48] Meissner H., Vacquier M., Kresse-Walczak K., Boening K. (2024). Mechanical, Optical and Surface Properties of 3D-Printed and Conventionally Processed Polyamide 12. Dent. Med. Probl..

[49] Wieckiewicz M., Opitz V., Richter G., Boening K.W. (2014). Physical Properties of Polyamide-12 versus PMMA Denture Base Material. BioMed Res. Int..

[50] Abdul-Monem M.M., Hanno K.I. (2024). Effect of Thermocycling on Surface Topography and Fracture Toughness of Milled and Additively Manufactured Denture Base Materials: An In-Vitro Study. BMC Oral Health.

[51] Sideridou I., Achilias D.S., Spyroudi C., Karabela M. (2004). Water Sorption Characteristics of Light-Cured Dental Resins and Composites Based on Bis-EMA/PCDMA. Biomaterials.

[52] Ferracane J.L., Berge H.X. (1995). Fracture Toughness of Experimental Dental Composites Aged in Ethanol. J. Dent. Res..

[53] Yiu C.K.Y., King N.M., Pashley D.H., Suh B.I., Carvalho R.M., Carrilho M.R.O., Tay F.R. (2004). Effect of Resin Hydrophilicity and Water Storage on Resin Strength. Biomaterials.

[54] Paradowska-Stolarz A., Wezgowiec J., Malysa A., Wieckiewicz M. (2023). Effects of Polishing and Artificial Aging on Mechanical Properties of Dental LT Clear^®^ Resin. J. Funct. Biomater..

[55] Aati S., Akram Z., Shrestha B., Patel J., Shih B., Shearston K., Ngo H., Fawzy A. (2022). Effect of Post-Curing Light Exposure Time on the Physico-Mechanical Properties and Cytotoxicity of 3D-Printed Denture Base Material. Dent. Mater..

[56] Paranna S., Thosar N., Kanitkar A. (2024). Effect of Build Orientation on Mechanical and Physical Properties of Additively Manufactured Resins Using Digital Light Processing Technology in Dentistry: A Systematic Review. J. Contemp. Dent. Pract..

[57] Alqutaibi A.Y., Aljohani R., Almuzaini S., Alghauli M.A. (2025). Physical-mechanical properties and accuracy of additively manufactured resin denture bases: Impact of printing orientation. J. Prosthodont. Res..

[58] Nam N.E., Hwangbo N.K., Jin G., Shim J.S., Kim J.E. (2023). Effects of heat-treatment methods on cytocompatibility and mechanical properties of dental products 3D-printed using photopolymerized resin. J. Prosthodont. Res..

[59] Nguyen J.F., Migonney V., Ruse N.D., Sadoun M. (2013). Properties of Experimental Urethane Dimethacrylate-Based Dental Resin Composite Blocks Obtained via Thermo-Polymerization under High Pressure. Dent. Mater..

[60] Kwaśny M., Bombalska A., Obroniecka K. (2022). A Reliable Method of Measuring the Conversion Degrees of Methacrylate Dental Resins. Sensors.

[61] Saini R.S., Vaddamanu S.K., Dermawan D., Mosaddad S.A., Heboyan A. (2024). Investigating the Role of Temperature and Moisture on the Degradation of 3D-Printed Polymethyl Methacrylate Dental Materials through Molecular Dynamics Simulations. Sci. Rep..

[62] Machado A.L., Puckett A.D., Breeding L.C., Wady A.F., Vergani C.E. (2012). Effect of thermocycling on the flexural and impact strength of urethane-based and high-impact denture base resins. Gerodontology.

[63] Arima T., Murata H., Hamada T. (1996). The effects of cross-linking agents on the water sorption and solubility characteristics of denture base resin. J. Oral Rehabil..

[64] Barszczewska-Rybarek I.M. (2019). A Guide through the Dental Dimethacrylate Polymer Network Structural Characterization and Interpretation of Physico-Mechanical Properties. Materials.

[65] Pomes B., Derue I., Lucas A., Nguyen J.-F., Richaud E. (2018). Water ageing of urethane dimethacrylate networks. Polym. Degrad. Stab..

[66] Greil V., Mayinger F., Reymus M., Stawarczyk B. (2023). Water sorption, water solubility, degree of conversion, elastic indentation modulus, edge chipping resistance and flexural strength of 3D-printed denture base resins. J. Mech. Behav. Biomed. Mater..

[67] Gad M.M., Alshehri S.Z., Alhamid S.A., Albarrak A., Khan S.Q., Alshahrani F.A., Alqarawi F.K. (2022). Water Sorption, Solubility, and Translucency of 3D-Printed Denture Base Resins. Dent. J..

[68] Carey B.J., Patra P.K., Ci L., Silva G.G., Ajayan P.M. (2011). Observation of Dynamic Strain Hardening in Polymer Nanocomposites. ACS Nano.

[69] Varol H.S., Meng F., Hosseinkhani B., Malm C., Bonn D., Bonn M., Zaccone A., Parekh S.H. (2017). Nanoparticle Amount, and Not Size, Determines Chain Alignment and Nonlinear Hardening in Polymer Nanocomposites. Proc. Natl. Acad. Sci. USA.

[70] Espíndola-Castro L.F., Durão M.A., Pereira T.V., Cordeiro A.B., Monteiro G.M. (2020). Evaluation of microhardness, sorption, solubility, and color stability of bulk fill resins: A comparative study. J. Clin. Exp. Dent..

[71] Takahashi Y., Hamanaka I., Shimizu H. (2013). Flexural properties of denture base resins subjected to long-term water immersion. Acta Odontol. Scand..

[72] Öztürk Z., Tosun B. (2025). Comparison of 3D Printed and Conventional Denture Base Materials in Terms of Durability and Performance Characteristics. Sci. Rep..

[73] Benli M., Al-Haj Husain N., Ozcan M. (2023). Mechanical and chemical characterization of contemporary occlusal splint materials fabricated with different methods: A systematic review. Clin. Oral Investig..

[74] Jindal P., Juneja M., Siena F.L., Bajaj D., Breedon P. (2019). Mechanical and Geometric Properties of Thermoformed and 3D Printed Clear Dental Aligners. Am. J. Orthod. Dentofac. Orthop..

[75] Weżgowiec J., Czapor-Irzabek H., Małysa A., Boening K., Kulbacka J., Więckiewicz M. (2025). Exploring the chemical stability and safety of intraoral appliances fabricated via 3D printing, thermoforming, and heat-activated polymerization. J. Prosthet. Dent..

[76] Gibreel M., Perea-Lowery L., Vallittu P.K., Lassila L. (2021). Characterization of occlusal splint materials: CAD-CAM versus conventional resins. J. Mech. Behav. Biomed. Mater..

[77] Spencer P., Ye Q., Park J., Topp E.M., Misra A., Marangos O., Wang Y., Bohaty B.S., Singh V., Sene F. (2010). Adhesive/Dentin Interface: The Weak Link in the Composite Restoration. Ann. Biomed. Eng..

[78] Grymak A., Aarts J.M., Ma S., Waddell J.N., Choi J.J.E. (2022). Wear Behavior of Occlusal Splint Materials Manufactured By Various Methods: A Systematic Review. J. Prosthodont..

[79] Özcan M., Jonasch M. (2018). Effect of Cyclic Fatigue Tests on Aging and Their Translational Implications for Survival of All-Ceramic Tooth-Borne Single Crowns and Fixed Dental Prostheses. J. Prosthodont..

